# Clinically relevant preservation conditions for mesenchymal stem/stromal cells derived from perinatal and adult tissue sources

**DOI:** 10.1111/jcmm.17016

**Published:** 2021-10-27

**Authors:** Anh T. L. Ngo, Hang M. Le, Nhung T. H. Trinh, Adriel Peng Guo Jun, Trung Q. Bach, Hue T. H. Bui, Van T. Hoang, Anh V. Bui, Liem T. Nguyen, Duc M. Hoang

**Affiliations:** ^1^ Vinmec Institute of Applied Science and Regenerative Medicine Vinmec Health Care System Hanoi Vietnam; ^2^ Vinmec Research Institute of Stem Cell and Gene Technology Vinmec Healthcare System Hanoi Vietnam

**Keywords:** adipose tissue, umbilical cord, bone marrow, cell therapy, mesenchymal stem/stromal cells, preservation condition, Ringer lactate, sodium chloride

## Abstract

The interplay between mesenchymal stem/stromal cells (MSCs) and preservation conditions is critical to maintain the viability and functionality of these cells before administration. We observed that Ringer lactate (RL) maintained high viability of bone marrow–derived MSCs for up to 72 h at room temperature (18°C–22°C), whereas adipose‐derived and umbilical cord‐derived MSCs showed the highest viability for 72 h at a cold temperature (4°C–8°C). These cells maintained their adherence ability with an improved recovery rate and metabolic profiles (glycolysis and mitochondrial respiration) similar to those of freshly harvested cells. Growth factor and cytokine analyses revealed that the preserved cells released substantial amounts of leukaemia inhibitory factors (LIFs), hepatocyte growth factor (HGF) and vascular endothelial growth factor‐A (VEGF‐A), as well as multiple cytokines (eg IL‐4, IL‐6, IL‐8, MPC‐1 and TNF‐α). Our data provide the simplest clinically relevant preservation conditions that maintain the viability, stemness and functionality of MSCs from perinatal and adult tissue sources.

## 1 INTRODUCTION

Mesenchymal stem/stromal cells (MSCs), first discovered in the 1960s, are a plastic‐adherent cell population possessing self‐renewal ability (limited *in vitro*) and differentiation potential into mesenchymal lineages, according to the International Society for Cell and Gene Therapy (ISCT).^1^, ^2^ In the last decade, MSC therapy has emerged as the most promising cell therapy due to its capabilities of *in vitro* expansion, direct interaction of MSCs with immunological cells to modulate the functions of the latter population, and secretion of growth factors and cytokines that promote cell survival, proliferation and function. MSCs for use in therapy including autologous and allogeneic administration originate from several sources, including adipose tissue (AD), bone marrow (BM), umbilical cord (UC), dental pulp, placenta and peripheral blood. In the last 25 years, more than 950 registered MSC‐based clinical trials have supported the safety profiles of treating over 10,000 patients with MSCs under clinical trial conditions,^3^ and 1,040 MSC‐based clinical trials targeting approximately 48,000 patients globally (searching term ‘mesenchymal stem cells’ at clinicaltrials.gov) have been registered to date.^4^ A recent study related to the production and application of MSCs in the United States revealed that AD‐, BM‐ and UC‐derived MSCs are the most widely used MSC sources for therapeutic applications and regenerative medicine.^5^ The lack of major histocompatibility (MHC) class II in combination with the expression of early embryonic surface markers in the naive form of these MSCs provides solid evidence for their multipotency and immunological privilege.^6^ Accessibility, easy and reproducible expansion, safety and potential effective treatment are considered major advantages of MSC therapy in various diseases, including orthopaedic injuries, cardiovascular disease,^7^ pulmonary disease (bronchopulmonary dysplasia),^8^, ^9^ graft‐vs.‐host disease^10^ and autoimmune disease,^11^ among others.

The majority of MSC products or therapies describe the use of cryopreservation conditions to store and transport the final product, which is usually thawed within a few hours prior to infusion. Many groups have identified challenges regarding the potential functionality of MSC products after preservation and thawing processes, particularly when bioactivity measurements are commonly conducted on MSCs before or without cryopreservation or following culture post‐thaw.^12^ The effects of storage and transport conditions, such as preservation solutions, temperature and duration of storage, play significant roles in cell viability and functionality. MSCs derived from various sources (such as AD‐, BM‐ and UC‐MSCs) might behave differently under these conditions, adding another layer of complexity to the quality control of stem cell therapies, especially in clinical trial settings. Hence, it is important to find the optimal conditions to maintain the quality of MSCs prior to performing clinical therapy while requiring minimal processing steps. Few studies have identified the optimal conditions to maintain the viability of AD‐,^13^ BM‐^14^ and UC‐MSCs.^14^ To our best knowledge, no study has reported the optimal conditions to maintain all three sources of MSCs, including AD‐, BM‐ and UC‐MSCs, for therapeutic applications.

**FIGURE 1:**
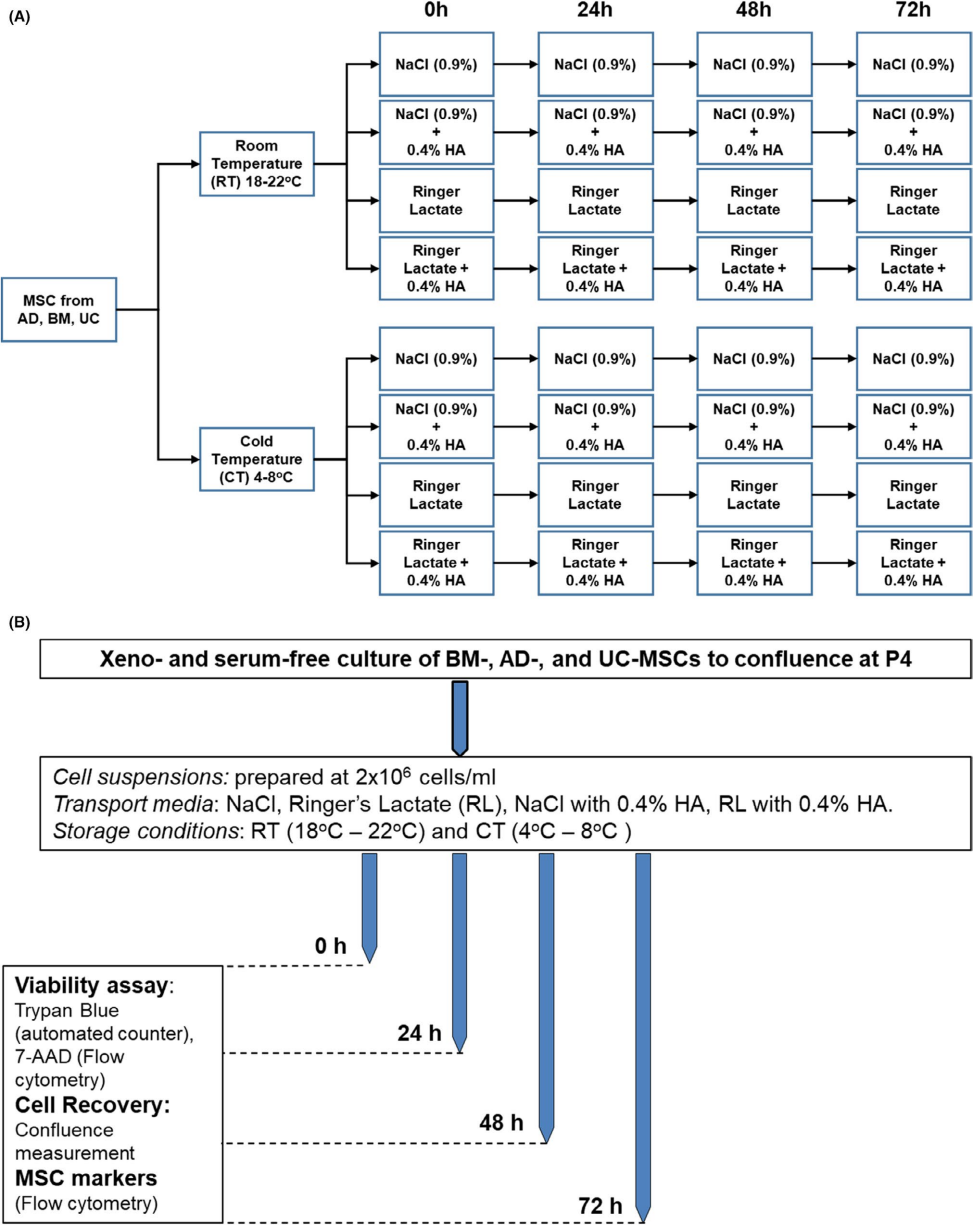
Experimental design and testing conditions. (A) hMSCs from AD, BM and UC tissues were cultured and suspended in preparation for various experiments in this study. Four transport media compositions and two transport temperatures (eight conditions per hMSC type, 32 total conditions) were prepared as follows: RL, RL supplemented with 0.4% HA, NaCl (0.9%), and NaCl (0.9%) supplemented with 0.4% HA; each of the media were stored at 4–8°C and 18–24°C. (B) Testing conditions of hMSCs at 0 h, 24 h, 48 h and 72 h after harvest to identify the optimal preservation conditions for MSCs from all three tested sources (AD, BM and UC)

Therefore, our study aimed to compare the effects of NaCl and Ringer Lactate (RL) and their combination with 0.4% human albumin (HA) on the viability, proliferation, marker expression, metabolic profile and paracrine functions of MSCs from three main sources: AD, BM and UC. The concentration of 0.4% HA was chosen because it has been used intensively as a supplement at our institute for preserving immune cells and is effective in maintaining the high quality of these cells before administration as a suspension solution.^15^, ^16^ We sought to determine how MSCs from these three sources behave under preservation conditions for up to 72 h and explore the metabolic activities and growth factor and cytokine secretion profiles from these cells before and after preservation; addressing these issues will allow us to identify suitable, clinically relevant preservation conditions for MSCs and support their therapeutic application in clinical trials. To this end, the experiments were designed to evaluate how MSCs derived from AD, BM and UC behave under different storage conditions and durations based on (1) cell viability (measured using 7‐ADD and Trypan blue), (2) cell proliferation and recovery, (3) MSC marker expression, (4) metabolic function and (5) analysis of growth factors/cytokines released from MSCs. Four transport/storage media, two temperature conditions and storage intervals up to 72 h were evaluated (Figure 1).

## 2 MATERIALS AND METHODS

### 2.1 Study approval

Adipose tissue, BM and UC tissues were obtained from healthy donors and collected at Vinmec International Hospital in 2019 after patients signed an informed consent form as previously reported.^17^ The collection of human samples was approved by the Ethics Board of Vinmec International Hospital (approval number: 122/2019/QD‐VMEC).

### 2.2 MSC culture and characterization

The three MSC lines from each respective tissue were randomly selected from the MSC biobank, were carefully characterized according to the ISCT guidelines, and were cultured under xeno‐free and serum‐free conditions as previously described.^17^ Culture reagents were purchased from Thermo Fisher Scientific (https://www.thermofisher.com/) and MACS Miltenyi Biotec (StemMACS^TM^ MSC expansion media kit XF, human, https://www.miltenyibiotec.com/) unless otherwise specified. To confirm the surface marker expression of the *in vitro*‐expanded cells isolated from AD, BM and UC, the harvested cells were subjected to flow cytometry analysis at P4 using a human BD Mesenchymal Stem kit (BD Biosciences; 562245) according to the manufacturer's protocol. Flow cytometry was performed using a BD FACSCanto flow cytometer (BD Biosciences). Data were analysed using FlowJo software (BD Biosciences). The trilineage differentiation assay was performed as previously described^17^ using a StemPro osteogenesis, adipogenesis and chondrogenesis differentiation kit (Gibco) according to the manufacturer's recommendation. The differentiated MSCs into osteogenic, adipogenic and chondrogenic lineages were visualized using Alizarin Red S, Oil Red O and Alcian Blue (Sigma‐Aldrich), respectively.

### 2.3 Evaluation of the effects of storage medium composition and temperature on stem cell quality

The MSCs from each respective tissue were thawed at P3 and cultured under xeno‐free and serum‐free conditions until 80% confluence followed by harvested using CTS^TM^ TrypLE^TM^ Select Enzyme (Gibco; A12859‐01). Next, the harvested MSCs (P4) were aliquoted into the 1.8 mL cryovials (Corning, New York, USA) to meet the requirement of a minimal cell infusion density of 2 × 10^6^ cells/mL. The MSCs were then resuspended in preservation media (NaCl, RL, NaCl+0.4%HA or RL+0.4%HA) and then stored at two different temperatures, 2°C–8°C and 18°C–22°C, without shaking and agitation. Three technical repeats per condition per tissue source were performed. The duration of storage was 24, 48 or 72 h. Cell viability was evaluated based on two independent methods, including an automated cell counter (Countess FL II, Thermo Fisher Scientific) using Trypan blue staining and flow cytometry measurement of 7‐AAD staining detected by a BD FACSCanto flow cytometer (BD Biosciences).

### 2.4 Cell recovery assessment

After subjecting the cells to the storage conditions in the four media and two temperature conditions, the cells were collected from the cryovials and plated into CTS CellStart‐coated 24‐well plates (Gibco; #A1014201) at a seeding density of 5,000 viable cells/cm^2^. Cell confluence was measured every 24 h using a Spark^®^ Multimode Microplate reader (Tecan) until the culture reached 80% confluence. The environmental settings were 37°C and 5% CO_2_ throughout the experimental process. The medium at 0, 24, 48 and 72 h was frozen for the analysis of growth factors and cytokines. Three technical repeats were performed for all the experiments.

### 2.5 Analysis of glycolysis and mitochondrial respiration

Cell mitochondrial stress and glycolytic stress were evaluated using an Agilent Seahorse XFe96 Analyzer (Agilent Technologies) according to the manufacturer's recommended protocol. Cells were seeded at 1 × 10^4^ cells per well onto Seahorse XF96 cell culture microplates (Agilent Technologies) coated with CTS^TM^ CellStart^TM^ and were incubated overnight at 37°C in 5% CO_2_. The XFe96 Extracellular Flux sensor cartridges (Agilent Technologies) were hydrated with distilled water and stored at 37°C without CO_2_ for 24 h and incubated with Agilent Seahorse XF Calibrant (pH 7.4) (Agilent Technology) for 1 h at 37°C without CO_2_ before the assay. On the day of the experiment, XF assay medium was supplemented with 10 mM glucose (Agilent Technologies, #103577‐100), 2 mM glutamine (Agilent Technologies, #103579‐100) and 1 mM pyruvate (Agilent Technologies, #103578‐100) and warmed to 37°C. The growth medium was removed, and the wells were rinsed with assay medium before they were then filled with 180 μL of assay medium; the cell culture plates were incubated for 1 h at 37°C without CO_2_.

The Seahorse XF Cell Glycolysis Stress test (Agilent Technologies, #103020‐100) used reagents at final concentrations of 10 mM glucose, 1.0 µM oligomycin and 50 mM 2‐DG per well. The Seahorse XF Cell Mito Stress test (Agilent Technologies, #103015‐100) used reagents at a final concentration of 1.0 µM oligomycin, 2.0 µM FCCP and 0.5 µM Rot/AA per well. The injection ports were loaded using the constant volume method described by the manufacturer. The plate was then loaded into the Agilent Seahorse XFe96 Analyzer, and the standard operating procedure for the Cell Mito Stress Test Assay was conducted following the manufacturer's recommendations.

After the assay was completed, the cells were fixed with 4% paraformaldehyde (PFA) and stained with DAPI staining solution (1:5000, Abcam; #ab228549). The DAPI signals were captured using the ImageXpress^®^ Micro Confocal High‐content Imaging System (Molecular Devices). The total cell number in each well was then calculated and used for normalization. At least three technical repeats were performed per storage condition for all the experiments.

### 2.6 Growth factor and cytokine quantification by multiplex immunoassay

Quantitative analysis of growth factors and cytokines was performed using the ProcartaPlex Human Immunoassay (Affymetrix, eBioscience). The supernatant from the MSCs used in the cell confluence experiment was collected on day five and stored at −80°C until further analysis. Samples were thawed on ice and subsequently centrifuged at 10,000 ×g for 10 min to remove particulates according to the manufacturer's protocol. We measured the levels of brain‐derived neurotrophic factor (BDNF), LIF, stem cell factor (SCF), vascular endothelial growth factor‐D (VEGF‐D), nerve growth factor‐beta (NGF‐β), epidermal growth factor (EGF), fibroblast growth factor 2 (FGF‐2), hepatocyte growth factor (HGF), platelet‐derived growth factor BB (PDGF‐BB), placenta growth factor‐1 (PIGF‐1) and vascular endothelial growth factor‐A (VEGF‐A) with the ProcartaPlex Human Growth Factors Panel 11plex (Thermo Fisher Scientific, EPX110‐12170–901) according to the manufacturer's instructions (MAN0016941). Human Custom ProcartaPlex 11‐plex (Thermo Fisher Scientific; PPX‐11‐MXNKTAD) was used to measure granulocyte‐macrophage colony‐stimulating growth factor (GM‐CSF), indoleamine 2,3‐dioxegenase (IDO), interferon‐ γ (IFN‐γ), interleukin‐10 (IL‐10), IL‐1β, IL‐4, IL‐6, IL‐8, monocyte chemoattractant protein 1 (MCP‐1), regulated upon activation, normal T cell expressed and presumably secreted (RANTES), and tumour necrosis factor‐α (TNF‐α) according to the manufacturer's instructions (MAN0017081). Plates were read in a Luminex 200 instrument (Luminex Corporation) controlled by xPONENT software and analysed on ProcartaPlexAnalyst software. Values after measurement were normalized to cell confluence, and values that were below the lowest limit of quantification (LLOQ) were assigned a value of 0.0 pg/mL. All samples were analysed in duplicate according to the manufacturer's suggestions.

### 2.7 Statistics

Statistical analyses were performed using two‐sided Student's *t* test with GraphPad Prism 8, unless otherwise stated. The data were presented as means ±SEMs. All the experiments were performed in biological triplicates with at least three technical repeats per biological sample for each individual tissue source. To compare the means of multiple groups, the data were analysed using two‐way ANOVA as indicated in the text. Significant differences in means are indicated as follows: *p < 0.05, **p < 0.01, ***p < 0.001, ****p < 0.0001.

## 3 RESULTS

### 3.1 Ringer lactate supports the survivability of MSCs derived from BM, AD and UC for up to 72 h

**FIGURE 2:**
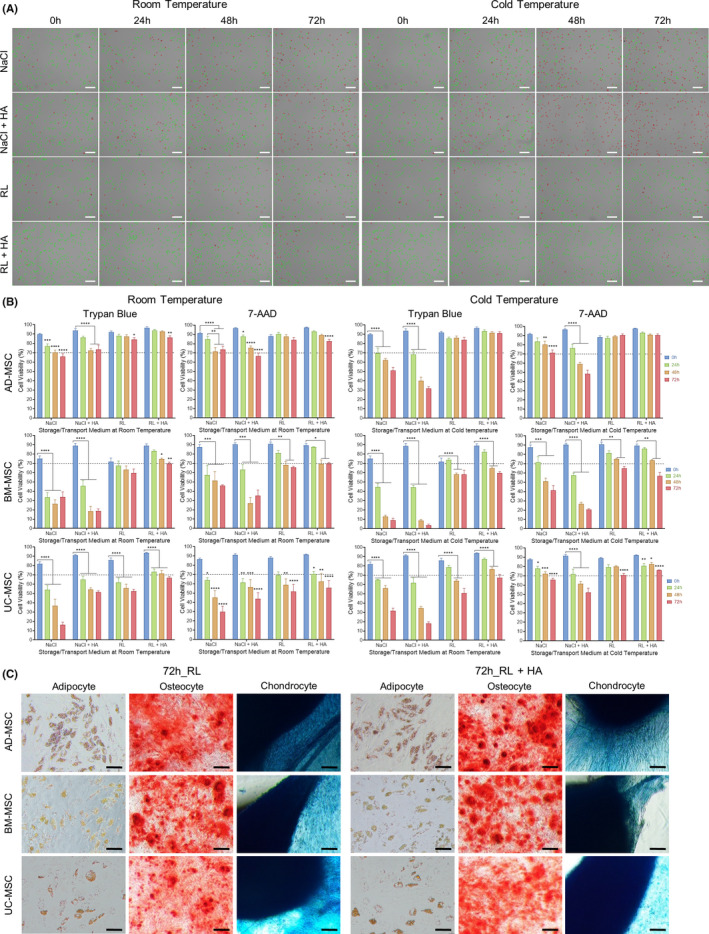
Cell viability assessment of MSCs from perinatal and adult sources under tested preservation conditions for up to 72 h. (A) Representative images of AD‐MSCs indicating live (green) and dead (red) cells under the tested preservation conditions using an automated cell counter at 0, 24, 48 and 72 h at either RT or CT. The images at 0 h timepoint of room temperature and cold temperature condition are identical to each other because of the same harvested cell source. (B) Comparative and quantitative analysis of cell viability of AD‐, BM‐ and UC‐MSCs in each temperature condition (grouped by preservation medium) was detected using Trypan blue staining and 7‐AAD (flow cytometry) (*n* = 3 per condition, mean ±SEM, *p < 0.05, **p < 0.01, ***p < 0.001, ****p < 0.0001 compared to all other conditions; one‐way ANOVA with Tukey's post hoc pairwise comparisons test).(C) Tri‐lineage differentiation potential of MSCs after 72 h storage in RL or RL supplemented with 0.4% HA at optimal storage temperature for each MSC type (ie cold temperature for UC‐, AD‐MSCs and room temperature for BM‐MSCs). *White scale bar*: *200 µm*, *Black scale bar*: *100 µm*

Mesenchymal stem/stromal cells from AD, BM and UC tissues were expanded *in vitro* to passage 4 (P4) and equally distributed into 4 preservation media followed by storage at either room temperature (RT, 18°C–22°C) or cold temperature (CT, 4°C–8°C) for 24 h, 48 h and 72 h as illustrated in Figure 1A. Cell viability, proliferation and the expression of MSC markers were measured as described in Figure 1B. To evaluate the impact of preservation conditions, cell viability was first analysed using Trypan blue staining (Table S1). Live and dead cells were counted using an automated cell counter (Figure 2A) and were confirmed by flow cytometry analysis of nucleic acid staining by 7‐AAD (Table S2). All data are presented as the means ±SEM with three biological replicates.

Harvested AD‐MSCs stored in NaCl‐based conditions showed a significant reduction in viability after 24 h in RT and CT (77% ± 2.5% and 70% ± 5.5%, respectively; *p* = 0.001). After 72 h of preservation, while 66% ± 3% of AD‐MSCs stored in NaCl at RT were viable, their CT counterparts showed a reduced viability to 62% ± 3%. The addition of 0.4% HA to NaCl did not significantly improve AD‐MSC viability after 24 h, 48 h and 72 h of storage. By contrast, RL‐based media supported the viability of AD‐MSCs in RT and CT for up to 72 h. At RT, RL‐ and RL+HA‐preserved AD‐MSCs exhibited 84% ± 2% and 86% ± 2% viability, respectively. Similar to the NaCl medium, 0.4% HA supplementation did not improve the viability of AD‐MSCs in all tested conditions (Figure 2B).

For BM‐MSCs, harvested cells stored in NaCl‐based medium in RT or CT exhibited a significant reduction in viability after 24 h (Figure 2B). After 48 h and 72 h, the viability of harvested BM‐MSCs decreased to below 40% under all NaCl‐based conditions regardless of storage temperature (Figure 2B, Table S1). Different behaviours of BM‐MSCs were observed under RL‐based conditions; in fact, BM‐MSCs preserved in either RL or RL+HA exhibited higher viability at RT than at CT. Even after 72 h of storage, BM‐MSCs preserved in RL+HA at RT showed 70% ± 1% viability, which is higher than that of their counterparts stored at CT (60% ± 1.5%; Figure 2B), suggesting that BM‐MSCs are sensitive to lower temperatures.

The behaviour of UC‐MSCs under different storage conditions was similar to that of AD‐MSCs. More specifically, NaCl‐based media did not support the viability of UC‐MSCs in either RT or CT. A significant decrease in cell viability in NaCl solutions was observed after 24 h at both tested temperatures and dropped dramatically after 72 h (Figure 2B, Table S1). Using RL‐based media improved the viability of UC‐MSCs, especially at CT (Figure 2B). UC‐MSCs maintained in RL+HA showed the highest survival rate after 72 h at CT (67% ± 4%) compared to other conditions.

Taken together, the results from the viability assays illustrated that the viabilities of AD‐, BM‐ and UC‐MSCs were best maintained in RL‐based media for up to 72 h (>70% viability) and that supplementation with 0.4% HA did not dramatically improve the viability of MSCs under the tested conditions. Interestingly, MSCs derived from BM exhibited the highest viability when stored at RT, whereas AD‐ and UC‐MSCs showed improved viability when stored at colder temperatures. Similar results were obtained in the 7‐AAD analysis, although the results showed a tendency of higher cell viability than that of the Trypan blue technique (Figure 2B, Table S2).

### 3.2 Distinct cell recovery profiles after 72 h of preservation

Cell attachment and proliferation are two important characteristics of MSCs that are directly affected by cell storage. To evaluate these two factors, dissociated MSCs in each condition were replated, and confluence was measured every 24 h until the cells reached 80% confluence. Table 1 presents the extracted data describing the preservation conditions allowing the stored cells to most rapidly reach 80%. Three distinct cell proliferation profiles were observed for MSCs derived from different origins, reflecting their recovery rate.

**TABLE 1:** Analysis of the recovery and proliferation capacity of AD‐, BM‐ and UC‐MSCs under the tested conditions for 24, 48 and 72 h (all: mean ± SEM, *n* = 3)

Sample name	Storage duration	Days until the first condition reached 80% confluence	Cell confluence (%)							
			Room temperature				4–8°C			
			NaCl	NaCl +0.4%HA	RL	RL +0.4%HA	NaCl	NaCl +0.4%HA	RL	RL +0.4%HA
AD‐MSCs	24 h	D5	77.89 ± 2.63	78.89 ± 1.89	81.33 ± 1.20	74.33 ± 5.84	78.56 ± 0.91	79.89 ± 0.99	81.56 ± 1.39	84.22 ± 2.51
	48 h		49.78 ± 12.81	71.11 ± 6.14	78.11 ± 3.07	81.11 ± 0.62	77.89 ± 0.91	80.11 ± 2.08	70.78 ± 6.45	82.22 ± 1.42
	72 h		2.33 ± 1.00	38.44 ± 12.83	75.89 ± 2.79	76.67 ± 3.83	76.83 ± 1.55	74.28 ± 4.87	69.00 ± 5.05	84.56 ± 0.73
BM‐MSCs	24 h	D6	50.56 ± 13.95	37.22 ± 15.17	75.56 ± 5.65	83.44 ± 2.06	70.33 ± 8.54	34.11 ± 13.39	41.44 ± 14.74	61.89 ± 6.71
	48 h	D8	15.11 ± 8.96	29.00 ± 9.51	67.78 ± 4.70	82.11 ± 0.11	69.67 ± 13.38	63.78 ± 14.39	42.67 ± 17.52	65.89 ± 4.04
	72 h	D8	6.22 ± 0.48	7.22 ± 1.06	65.33 ± 8.28	80.22 ± 0.40	45.67 ± 17.13	32.00 ± 4.60	33.33 ± 14.44	54.00 ± 7.69
UC‐MSCs	24 h	D5	29.00 ± 4.53	56.11 ± 13.25	61.33 ± 13.77	72.44 ± 6.41	84.67 ± 1.35	74.56 ± 5.39	77.44 ± 3.07	88.22 ± 0.68
	48 h		3.56 ± 1.31	13.67 ± 5.17	26.67 ± 11.61	36.22 ± 14.23	69.44 ± 3.23	66.22 ± 10.13	52.67 ± 8.06	83.44 ± 2.47
	72 h		1.89 ± 1.57	1.78 ± 0.89	14.89 ± 13.58	23.56 ± 19.69	68.78 ± 9.58	72.78 ± 5.94	39.89 ± 12.63	86.00 ± 2.52

Compared with BM‐MSCs, AD‐MSCs and UC‐MSCs exhibited the most consistent and rapid recovery cell type after preservation. After 24 h of storage, AD‐MSCs under all tested conditions only required 5 days to reach 80% confluence regardless of the base medium and storage temperature. Forty‐eight hours of preservation revealed variations in which cells kept in NaCl‐based media had a reduced recovery rate at RT, whereas cells in RL‐based media still reached 80% confluence within 5 days. After 72 h of preservation in RL+0.4% HA at CT, AD‐MSCs maintained a recovery rate of 5 days to reach 80% confluence, whereas cells stored in NaCl at RT could not attach and proliferate (Table 1; Figure S1).

Storage conditions, duration and temperature exhibited a great impact on the BM‐MSC recovery rate. After 24 h of preservation, cells maintained in NaCl at CT or in RL or RL+0.4%HA at RT showed a better recovery rate; cells stored in RL+0.4%HA at RT reached 80% confluence after 6 days. Similar results were observed when BM‐MSCs were maintained for 48 h and 72 h. RL‐based media supported BM‐MSCs better than did NaCl‐based media in terms of recovery rate. BM‐MSCs stored in RL+0.4% at RT for 48 h and 72 h required 8 days to reach 80% confluence, whereas their NaCl‐based counterparts could not attach and proliferate (Table 1; Figure S1).

The sensitivities of UC‐MSCs under different preservation conditions were also observed (Table 1). In general, storage temperature and RL‐based media play a major role in the recovery rate of UC‐MSCs. After 24 h of preservation, while cells in NaCl and NaCl+0.4% HA at RT exhibited a slow recovery rate, their counterparts in CT showed faster proliferation and required only 5 days to reach 80% confluence (Table 1). Cells maintained in RL‐based media demonstrated a similar recovery rate, in which cells stored in RL+0.4%HA at CT reached 80% confluence within 5 days. UC‐MSCs stored for prolonged durations of 48 h and 72 h exhibited a temperature‐dependent proliferation rate in NaCl‐based and RL‐based media, in which RL‐based media at CT strongly maintained the proliferation rate of UC‐MSCs. Interestingly, cells preserved in RL+0.4%HA at CT for 48 h and 72 h could still reach 80% confluence after 5 days of culture post‐preservation (Table 1; Figure S1).

Taken together, our results demonstrated that the recovery rate of MSCs derived from different sources is strongly affected by storage media, temperature and duration. Among all tested cells, AD‐MSCs were the most resilient cell type and are less affected by all tested conditions than were BM‐MSCs and UC‐MSCs, whereas the recovery rate of BM‐MSCs is strongly altered by the storage conditions. UC‐MSCs are relatively more sensitive to temperature, especially during prolonged storage of up to 72 h. These results together with the viability measurements provided sufficient data to suggest that RL‐based media is more suitable for the preservation of AD‐, BM‐ and UC‐MSCs than NaCl‐based media under the tested conditions.

### 3.3 Evaluation of MSC surface markers and differentiation potential of AD‐, BM‐ and UC‐MSCs under different storage conditions

To confirm MSC identity and the effects of storage conditions on MSC surface marker expression, we performed flow cytometry analysis of the expression of MSC‐positive and MSC‐negative markers according to ISCT guidelines. Our results revealed that all tested hMSCs steadily maintained CD73 and CD90 expression at 0 h and 72 h storage at RT and CT in the tested medium (Table 2; Figure S2). Although there was a small fluctuation in CD105 expression observed after 72 h of storage in all tested media and temperatures, the expression was higher than 95% (Table 2; Figure S2). The expression of negative markers, including CD11b, CD19, CD34, CD45 and HLA‐DR, remained below 2% regardless of storage medium, temperature and storage duration. These expression levels met the recommendation of the ISCT for MSC identification evaluation.

**TABLE 2:** Surface marker profiles of AD‐, BM‐ and UC‐MSCs at 0 and 72 h maintained at Room temperature and a cold temperature under different storage conditions (all: mean ±SEM, *n* = 3)

Sample name	Surface markers	0 h	72 h							
			Room temperature				Cold temperature			
			NaCl	NaCl+0.4%HA	RL	RL+0.4%HA	NaCl	NaCl+0.4%HA	RL	RL+0.4%HA
AD	CD90+	99.97 ± 0.06	99.47 ± 0.92	99.97 ± 0.06	99.93 ± 0.12	99.93 ± 0.06	99.97 ± 0.06	99.97 ± 0.06	99.97 ± 0.06	99.97 ± 0.06
	CD73+	99.93 ± 0.12	99.40 ± 0.10	99.63 ± 0.21	99.50 ± 0.46	99.47 ± 0.48	99.80 ± 0.10	99.80 ± 0.10	99.63 ± 0.25	99.77 ± 0.15
	CD105+	99.8 ± 0.17	93.03 ± 2.34	96.50 ± 2.04	96.90 ± 2.10	97.47 ± 1.78	98.57 ± 1.57	98.7 ± 0.95	97.47 ± 1.85	98.37 ± 0.95
	Neg markers	1.22 ± 0.55	0.38 ± 0.32	0.27 ± 0.28	0.27 ± 0.22	0.27 ± 0.20	0.27 ± 0.17	0.27 ± 0.17	0.24 ± 0.14	0.24 ± 0.15
UC	CD90+	98.77 ± 1.29	99.23 ± 0.15	99.37 ± 0.46	99.17 ± 0.71	99.47 ± 0.59	99.53 ± 0.35	99.80 ± 0.10	99.47 ± 0.45	99.50 ± 0.69
	CD73+	99.60 ± 0.10	98.07 ± 2.48	99.37 ± 0.23	99.40 ± 0.56	99.60 ± 0.30	99.93 ± 0.06	99.90 ± 0.10	99.80 ± 0.20	99.90 ± 0.10
	CD105+	98.90 ± 1.04	96.67 ± 1.44	96.03 ± 1.65	97.10 ± 0.62	96.90 ± 1.65	98.20 ± 1.05	98.03 ± 1.60	97.03 ± 1.31	97.5 ± 1.81
	Neg markers	0.08 ± 0.08	0.33 ± 0.35	0.12 ± 0.15	0.03 ± 0.01	0.11 ± 0.13	0.13 ± 0.08	1.00 ± 1.32	0.21 ± 0.28	0.20 ± 0.32
BM	CD90+	100 ± 0.00	98.63 ± 1.46	98.8 ± 1.13	99.57 ± 0.49	99.83 ± 0.12	99.33 ± 0.72	99.63 ± 0.38	99.90 ± 0.10	99.9 ± 0.00
	CD73+	99.23 ± 1.33	97.80 ± 2.43	97.03 ± 2.35	99.60 ± 0.26	99.67 ± 0.06	97.73 ± 1.89	98.93 ± 1.00	99.20 ± 0.40	99.83 ± 0.06
	CD105+	99.87 ± 0.23	95.80 ± 2.88	97.83 ± 1.07	97.03 ± 0.74	97.70 ± 1.18	97.60 ± 2.39	97.37 ± 2.72	97.57 ± 1.38	98.83 ± 1.33
	Neg markers	0.38 ± 0.60	0.71 ± 0.72	1.46 ± 1.14	0.65 ± 0.52	0.51 ± 0.78	0.53 ± 0.89	0.56 ± 0.43	0.36 ± 0.53	1.05 ± 1.18

To investigate the effect of storage conditions on MSC differentiation ability (AD and UC‐MSCs were stored at CT and BM‐MSCs were stored at RT), all MSC types were preserved in RL and RL supplemented with 0.4% HA at the optimum storage temperatures for each cell type. The results showed that all tested MSCs were able to differentiate into osteogenic, adipogenic and chondrogenic lineages, with no difference between before and after storage, or between RL and RL supplemented with 0.4% HA. (Figure 2C and Figure S3). Consequently, MSCs preserve their fundamental biological features after 72 h of storage in RL‐based media at optimum temperature, as recommended by ISCT.

### 3.4 Metabolic analysis of hMSCs under different storage conditions

To determine whether the survival and recovery rate in hMSCs was accompanied by changes in metabolism, we examined the two major metabolic pathways in the cells glycolysis and mitochondrial respiration with a Seahorse XF‐96 analyzer. Based on the above analysis, cells that maintained the selected storage conditions for BM‐MSCs (RL+HA at RT) and AD‐ and UC‐MSCs (RL+HA at CT) at 72 h were directly compared to those stored under three suboptimal conditions, and freshly harvested cells (0 h) were used as the positive control (Table 3). We were unable to perform the experiment for UC‐MSCs maintained in the suboptimal condition due to the high rates of cell death and the low number of cells attached to the Seahorse cell culture plate after 24 h.

**TABLE 3:** Suggested preservation conditions for metabolic analysis and measurement of growth factors and cytokines

Cell sources	Preservation conditions	
	Suggested condition (72 h best)	Worse condition (72 h worse)
AD‐MSCs	Ringer lactate +0.4% HA at cold temperature (4°C–8°C)	NaCl +0.4% HA at cold temperature (4°C–8°C)
BM‐MSCs	Ringer lactate +0.4% HA at room temperature (18°C–22°C)	NaCl +0.4% HA at cold temperature (4°C–8°C)
UC‐MSCs	Ringer lactate +0.4% HA at cold temperature (4°C–8°C)	NaCl at room temperature (18°C–22°C)

**FIGURE 3:**
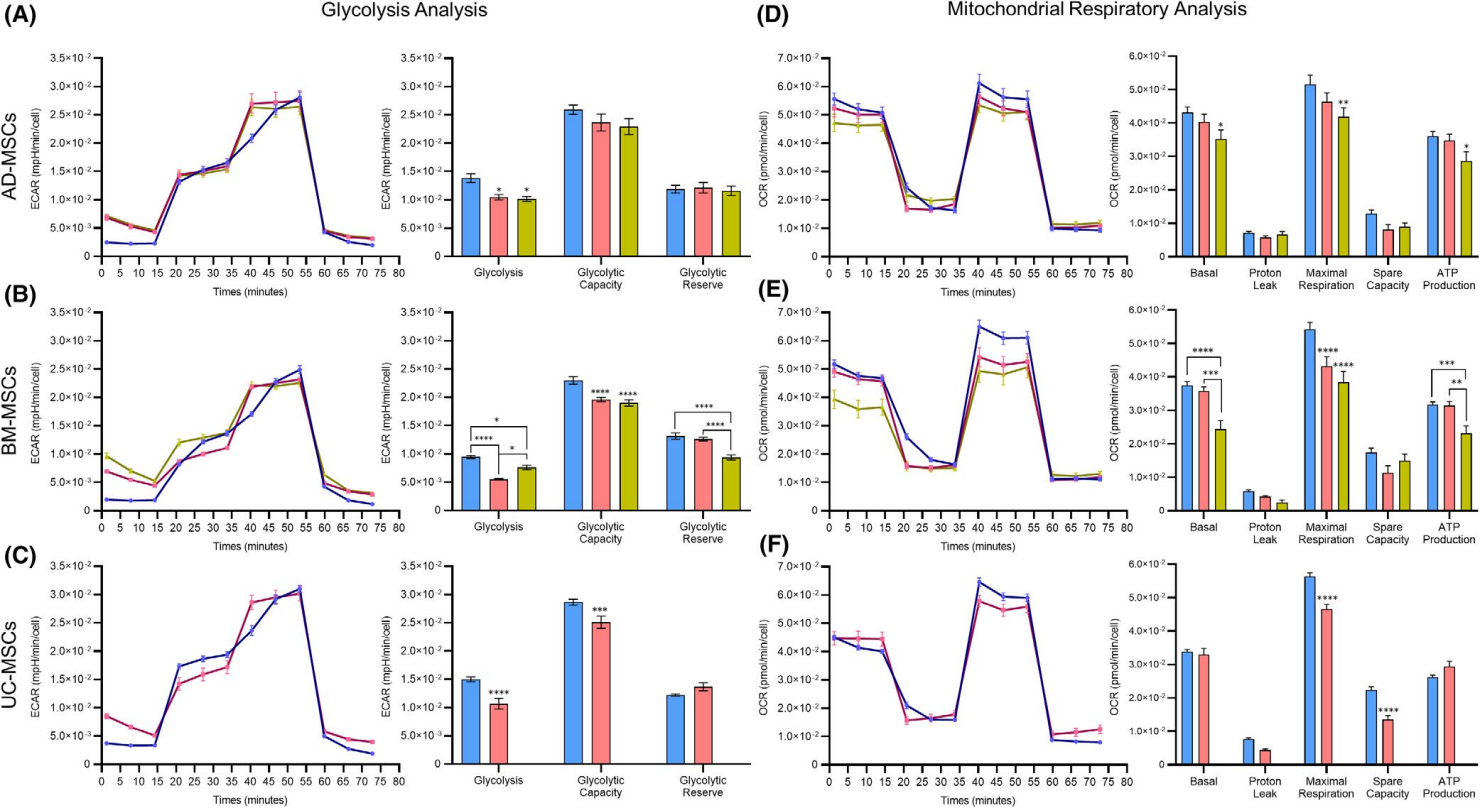
Metabolic analysis of AD‐, BM‐ and UC‐MSCs preserved under optimal conditions (BM‐MSCs (RL+HA at RT), AD‐ and UC‐MSCs (RL+HA at CT) at 72 h) and suboptimal conditions as well as their freshly harvested counterparts. Glycolysis analysis of AD‐MSCs (A), BM‐MSCs (B) and UC‐MSCs (C) using the Seahorse XF Glycolysis Stress Test Kits; arrows indicate the injection of glucose (Glu.), oligomycin (Olig.) and 2‐deoxyglucose (2‐DG) into the assay media. The calculated glycolysis activity, glycolytic capacity and glycolytic reserves of all three tested MSCs are shown in the bar chart. Mitochondrial respiration was measured using the Seahorse XF Cell Mito Stress Test for AD‐MSCs (D), BM‐MSCs (E) and UC‐MSCs (F). Arrows indicate injections of the specific stressors oligomycin (Olig.), carbonyl cyanite‐4 phenylhydrazone (FCCP) and rotenone/antimycin A (Rot.) into the assay media (*n* = 3 per condition; mean ±SEM; *p < 0.05, **p < 0.01, ***p < 0.001, ****p < 0.0001; two‐way ANOVA with Tukey's multiple comparisons test for AD‐MSCs and BM‐MSCs and Šídák's multiple comparisons test for UC‐MSCs)

In terms of glycolysis analysis, MSCs derived from all three tested sources exhibited a significant reduction in basal glycolysis at 72 h under the optimal and suboptimal conditions compared to their counterparts before storage (0 h) (Figure 3A,B,C). No significant difference in glycolytic capacity or glycolytic reserve in AD‐MSCs was found between the tested conditions (Figure 3A). The glycolysis analysis of BM‐MSCs revealed that the glycolytic capacity was reduced after preservation for 72 h in the optimal and suboptimal conditions. Moreover, BM‐MSCs showed a significant reduction in glycolytic reserve when they were stored in suboptimal conditions compared to that of cells stored in optimal conditions and positive control cells (Figure 3B). UC‐MSCs followed similar patterns to BM‐MSCs, where a significant decrease in glycolytic capacity was observed when the cells were stored for 72 h under the optimal conditions, and their glycolytic reserve was similar to that of the positive control cells (Figure 3C).

To assess the mitochondrial function of AD‐, BM‐ and UC‐MSCs in the optimal storage conditions, a Cell Mito Stress test was performed on the optimal, suboptimal and freshly harvested cells. The Seahorse XF Cell Mito Stress test showed that all three MSC populations exhibited comparable basal mitochondrial respiration parameters between the control and the optimal storage conditions, whereas cells stored in the suboptimal conditions showed a significant drop in their basal respiratory level (Figure 3D,E,F). A significant decrease in maximal respiration was observed in BM‐ and UC‐MSCs maintained in the optimal and suboptimal conditions compared to that of the control group (Figure 3E,F). AD‐MSCs reduced their maximal respiration after 72 h of maintenance in the worst conditions compared to the control group. Increased ATP production was observed in AD‐, BM‐ and UC‐MSCs stored in optimal conditions for 72 h, whereas AD‐ and BM‐MSCs showed a reduction in ATP production in the suboptimal conditions.

### 3.5 Analysis of growth factors and cytokines released from AD‐, BM‐ and UC‐MSCs under different preservation conditions

**FIGURE 4:**
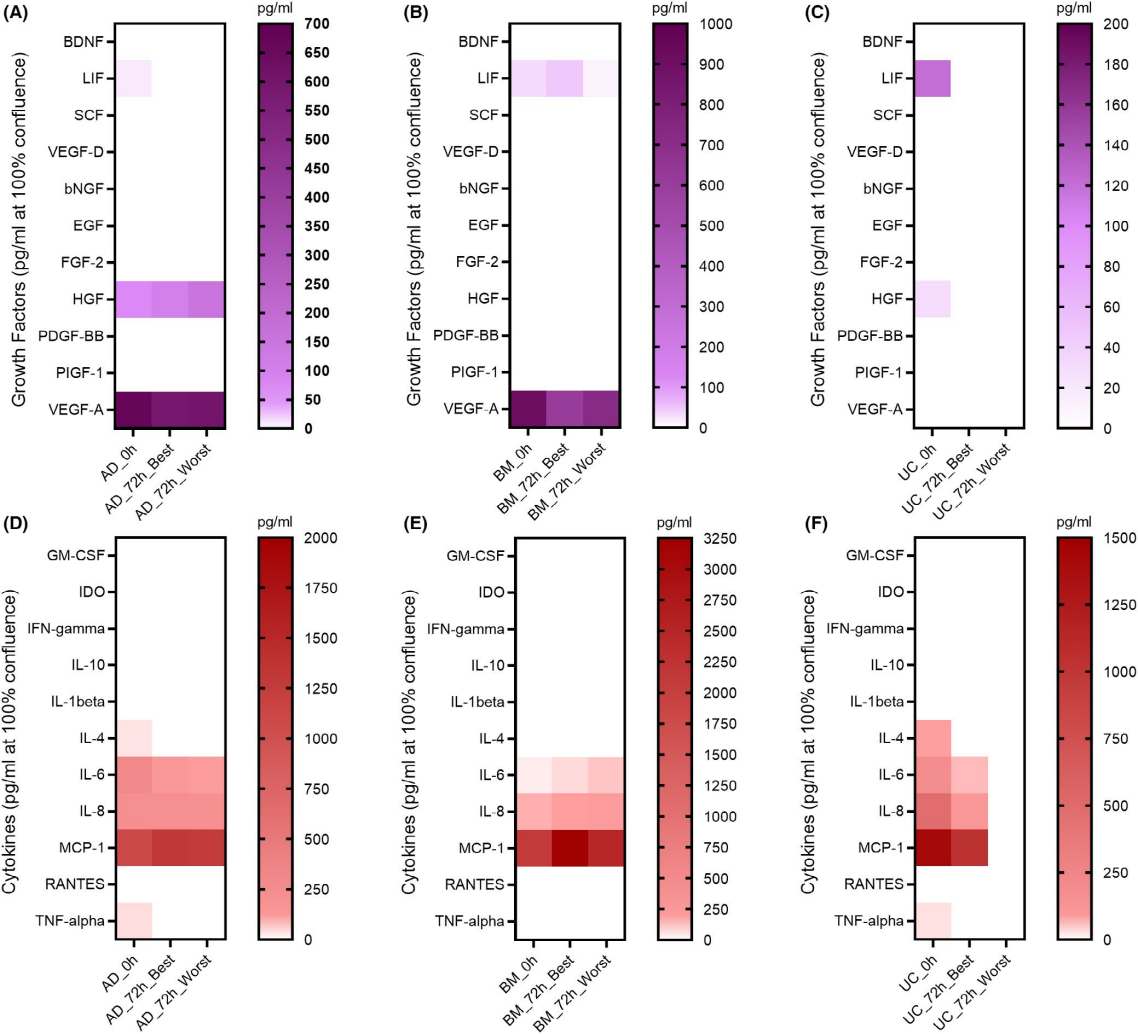
Paracrine analysis of growth factors and cytokines released from AD‐, BM‐ and UC‐MSCs preserved under optimal and suboptimal conditions and as well as from their freshly harvested counterparts. Eleven growth factors reported to be commonly released from AD‐MSCs (A), BM‐MSCs (B) and UC‐MSCs (C) were measured when these cells were stored under the tested conditions. Eleven cytokines reported to be secreted from AD‐MSCs (D), BM‐MSCs (E) and UC‐MSCs (F) under the tested preservation conditions were measured. The concentrations of growth factors and cytokines were calculated (pg/mL) and normalized based on the cell confluence results obtained from a Tecan Multimode plate reader via a cell confluent measurement assay

To determine whether the preservation conditions altered the level of paracrine factors released by MSCs, culture media collected from cells subjected to each storage condition were subjected to Luminex technology in order to identify the presence of growth factors and cytokines (Table S3). Among the 11 tested growth factors, LIF, HGF and VEGF‐A were detectable in freshly harvested AD‐MSCs, whereas LIF and VEGF‐A were found in media derived from BM‐MSCs (Figure 4A,B). UC‐MSCs were found to secrete LIF and HGF (Figure 4C). Under the selected conditions, AD‐MSCs and UC‐MSCs stopped secreting LIF, while the LIF level was increased in medium collected from BM‐MSCs. HGF levels were increased in medium collected from AD‐MSCs but were not detected in either BM‐ or UC‐MSCs preserved under either the optimal or suboptimal conditions. A reduction in VEGF‐A levels was detected in AD‐ and BM‐MSCs in all preservation conditions. In terms of cytokine secretion analysis, among the 11 tested cytokines, IL‐4, IL‐6, IL‐8, MCP‐1 and tumour necrosis factor alpha (TNF‐α) were found to be secreted from AD‐, BM‐ (except IL‐4 and TNF‐α) and UC‐MSCs under normal culture conditions (Figure 4D,E,F, respectively). Under the tested preservation conditions, IL‐4 and TNF‐α secretion from AD‐ and UC‐MSCs was diminished. Although IL‐6 levels were reduced when AD‐ and UC‐MSCs were preserved under the optimal and suboptimal conditions, they were increased in BM‐MSCs. IL‐8 and MCP‐1 levels were found to be increased in AD‐ and BM‐MSCs when they were preserved in the optimal and suboptimal conditions compared to their freshly cultured counterparts, whereas UC‐MSCs reduced the secretion of both cytokines under the tested conditions.

## 4 DISCUSSION

With the rapid development of cell‐based regenerative therapy for clinical application, it has become more crucial to establish preservation conditions that allow maintenance of cell quality prior to patient delivery. Although it is better to administer cells immediately after harvest, *in vitro* cultured MSCs are usually kept for hours or days for final quality control evaluations (such as microorganism testing at harvest time, which takes at least 24 h for inoculation). Moreover, potential challenges have been posed during the transportation process of cell products from manufacturing sites to the bedside and have drawn great attention in regenerative medicine. Our study successfully identified acceptable and clinically relevant preservation conditions for MSCs derived from the three most commonly used tissues (AD, BM and UC), as shown in Table 3. Moreover, the results demonstrated that the storage medium, temperature and duration had profound effects on the survival, recovery rate, metabolic function and paracrine secretion of MSCs regardless of their origin. Although various transportation strategies have been widely investigated and numerous studies have been conducted to understand the effects of preservation conditions on cell quality, our study provides simple conditions for maintaining MSCs at an acceptable level of quality for a maximum of 72 h without the need for cryo‐protectants, allowing the direct administration of these cells. In our study, we noted a significant difference in viability measurement using Trypan blue and 7‐AAD, which was in line with a previous study.^18^ Our observation is not unexpected, as the detection of cell viability via membrane integrity frequently does not correlate with functional measures of viability.^19^

Different types of preservation media have been tested previously, such as PBS, M199, culture medium, plasma lysate A, DMEM supplemented with 1% HSA, NaCl and RL.^20^ However, the use of PBS, M199, culture media and DMEM are inappropriate for clinical and therapeutic treatment because they are not approved vehicles for safe infusion into patients. Therefore, in our study, NaCl and RL were chosen as the main base media for MSC preservation because they have been widely used in numerous clinical trials.^3^, ^8^, ^21^ It was reported that MSCs maintained in saline or dextrose for more than 2 h exhibited significantly reductions in viability, CFU capacity and differentiation ability.^22^ Based on the theory of cell ion and osmotic homeostasis, cell viability is strongly regulated by the osmolality and electrolyte concentration of the extracellular environment.^23^ The nature of saline is isotonic, containing only sodium ions and chloride ions, whereas RL solution is a mixture of not only sodium and chloride ions but also potassium ions and calcium ions, mimicking the extracellular fluid in the human body. Our results support the notion that Ringer's mixture of ions maintains high cell viability, cell attachment and recovery after 72 h of storage. This was consistent with a previous study reporting that the ions in RL prevented cell death and accounted for the differences between normal saline and RL solution in the regulation of cell viability.^24^ Several mechanisms are found to support the protective functions of RL on cell viability, which are linked to the extracellular concentrations of Na^+^, K^+^ and Ca^2+^. Recent studies indicate that inhibition of the Na^+^/K^+^‐ATPase pump in low K^+^ medium resulted in a reduction in intracellular K^+^ concentration, which, in turn, slowed the proliferation rate of cell lines *in vitro*.^25^, ^26^ It was reported that a K^+^ concentration of approximately 500 µmole/g protein is the threshold by which it would directly prevent the proliferation process.^27^, ^28^ Regarding the MSC proliferation potential, recent research suggests that the growth‐dependent cell K^+^ concentrations dropped within the range between 600 and 1,100 µmol/g protein, which was associated with the accumulation of G1 cells in the population and resulted in reduced proliferation and delays in cell cycle progression.^29^ Similarly, it is well known that Ca^2+^ plays a significant role in stem cell proliferation, differentiation and apoptosis.^30^ A recent study reported that elevated extracellular Ca^2+^ concentrations promoted MSC proliferation and attachment by enhancing extracellular matrix mineralization function.^31^ This result explains and supports our observation that MSCs derived from the three tested sources exhibited better attachment and recovery rates when they were preserved in RL‐based medium than in saline‐based solutions. Finally, in our study, supplementation of 0.4% HA to preservation‐based medium slightly improved the viability of MSCs for up to 72 h. The concentration of HA used in our study was adapted from a previously described immunological cell therapy.^15^ It is important to note that MSCs stored in RL+0.4% HA showed a better recovery rate and stable proliferation during the replating process. Taken together, our results confirm that RL‐based medium is suitable to support the viability, proliferation and recovery of AD‐, BM‐ and UC‐MSCs.

Regarding storage temperature, our results showed that AD‐ and UC‐MSCs maintained high viability levels (more than 80%) under CT, whereas BM‐MSCs showed significantly reduced viability when maintained under low temperatures. The temperature‐dependent viability of MSCs derived from various sources has been confirmed by many authors and thoroughly reviewed.^32^, ^33^, ^34^ A low survival rate and recovery rate of <25% of cells were previously observed in BM‐MSCs stored in RL solution or Plasma‐Lyte^®^ at CT.^35^ Similar results have also been reported, where the viability of MSCs stored at 4°C decreased by 15% in saline and Plasma‐Lyte A solution.^36^ Interestingly, viability <50% was reported in placenta‐derived MSCs stored for 72 h compared with that in the non‐stored control.^37^ Hence, our data demonstrate that RL+0.4% HA provides an acceptable condition for AD‐ and UC‐MSCs preservation at CTs, whereas BM‐MSCs exhibited a better survival rate and recovery capacity at RT. Another discussion point is the maximum duration by which the cells could maintain their viability and function. In the present study, the optimal conditions for each MSC type support viability up to 72 h, although we believe that 24 h was sufficient to deliver the cell products to another city across the country and prepare the final QC results according to the release criteria before patient administration.

Since our data revealed RL as a universally clinically relevant preservation medium for AD‐, BM‐ and UC‐MSCs, how these MSCs maintain their markers, metabolic activities and paracrine functions *in vitro* must be investigated, and hopefully will reflect the potential behaviour of these cells after patient administration. Consistent with other studies, our results demonstrate that the preservation conditions for short‐term storage do not alter the common positive MSC markers (CD73, CD90 and CD105); furthermore, under optimum storage conditions, MSC trilineage differentiation capacity is also maintained, suggesting that MSC products meet the recommendations by the ISCT.^38^, ^39^, ^40^ To evaluate the metabolic function of preserved MSCs, glycolysis and mitochondrial respiration were measured in preserved cells after they were replated and cultured for 24 h. Our results showed a decrease in basal glycolysis and mitochondrial respiration in MSCs from all tested sources after 72 h of preservation under optimal conditions. A similar phenomenon was also reported by Killer et al., who illustrated that the metabolic activity of MSCs increases upon thawing.^41^ Antebi et al. described a decrease in the metabolic activity of freshly thawed MSCs, which later recovered after a 24 h acclimation period.^38^ Interestingly, for the first time, our data revealed a significant reduction in maximal respiration of MSCs after 72 h of preservation under suboptimal conditions compared to that of their freshly cultured and optimally stored counterparts. In addition to their natural function as the energy factory of cells, mitochondria also serve as crucial regulators of apoptosis when cells experience stress or injury.^42^, ^43^ Upon encountering severe stress caused by the preservation process, the normal homeostasis of mitochondria is compromised, resulting in mitochondrial dysfunction and consequent cell death. Although our study did not investigate the molecules underlying the observed mitochondrial stress, several studies have been performed to address this problem. One group showed that cryopreservation and serum deprivation trigger mitochondrial stress, leading to increased intracellular reactive oxygen species (ROS) levels, impaired mitochondrial membrane permeability (MMP) and cytochrome c release.^44^, ^45^, ^46^ Under preservation stimuli such as nutrient starvation and biological waste accumulation, functional disruption of the mitochondrial machinery subsequently generates excessive intracellular ROS, a key component involved in undesirable oxidative DNA damage, diminished protein production processes, and intracellular organelle dysfunction.^47^, ^48^, ^49^

An attractive aspect of MSCs in regenerative therapy is their immunomodulatory response to stimulation from the surrounding environment via secretion of a wide range of beneficial growth factors and cytokines. We used Luminex technology to investigate the secreted bioactive factors in the conditioned media from MSCs maintained under different preservation conditions. Our results showed that MSCs from different sources were able to secrete LIF, HGF, VEGF‐A, IL‐4, IL‐6, IL‐8, MCP‐1 and TNF‐α when they were freshly cultured in xeno‐free and serum‐free conditions. It is well known that MSCs can be activated by adding inflammatory cytokines such as interferon‐γ (IFN‐γ) alone or in combination with TNF‐α or IL‐1. Under xeno‐free and serum‐free conditions, MSCs derived from AD, BM and UC tissues secreted substantial amounts of leukaemia inhibitory factor (LIF), whereas AD‐ and UC‐MSCs produced hepatocyte growth factor (HGF) and TNF‐α. LIF, HGF and TNF‐α are the three potent immunosuppressive factors that are commonly reported to be associated with the therapeutic effects of MSC therapy.^50^, ^51^, ^52^ More importantly, it was suggested that the secretion of LIF from MSCs is a predictor and indicator to confirm the progenitor nature of MSCs.^53^ AD‐ and BM‐MSCs in our culture conditions also secreted high levels of VEGF‐A, which is consistent with previous studies reporting the involvement of upregulated VEGF gene expression in AD‐ and BM‐MSCs in the regulation of MSC proliferation.^54^, ^55^ All three sources of tested MSCs also produced IL‐4 (except for BM‐MSCs), IL‐6, IL‐8 and MCP‐1. IL‐6 was first described in 1986 as a stimulatory factor of B lymphocytes^56^ and was reposted to be involved in the orchestration of the immune response, hematopoiesis, inflammation, cell survival, apoptosis, cell proliferation and oncogenesis.^56^, ^57^ In concert with TNF‐α and IL‐4, IL‐6 induces the secretion of acute‐phase proteins, triggering the recruitment of neutrophils and their conversion to macrophages to induce inflammation as well as stimulating T cell proliferation.^58^, ^59^ Notably, the preservation conditions directly altered the secretory profiles of MSCs from all the tested sources, and their behaviour in response to the surrounding environment was also distinct. While AD‐ and UC‐MSCs preserved in either optimal or suboptimal conditions did not release LIF into the culture medium, BM‐MSCs released significantly higher levels of LIF in the optimal condition than did their freshly cultured counterparts. IL‐4, IL‐6 and IL‐8 concentrations followed the same pattern as LIF levels, whereas MCP‐1 levels were increased in AD‐ and BM‐MSCs and decreased in UC‐MSCs in both tested conditions. Our results support a previous study reporting that the upregulation of LIF (twofold to 90‐fold) and IL‐6 (twofold to 150‐fold) expression was detected in MSCs under fluid shear stress conditions.^60^

In conclusion, our study presents simple and clinically relevant preservation conditions for MSCs derived from the three most common sources (AD‐, BM‐ and UC‐MSCs) for clinical application; these conditions maintain the stemness features and functionality after up to 72 h of storage. Our results show that AD‐, BM‐ and UC‐MSCs could be stored for 72 h at 18°C–22°C (for BM‐MSCs) or at 4°C–8°C (for AD‐ and UC‐MSCs) in RL or RL+HA solution. We also analysed the phenotype, metabolism and paracrine profile of MSCs after they were stored under the aforementioned conditions, providing a basis for future studies to investigate the potential mechanism that enhances the effectiveness of preserved MSC therapy. As elucidated in this study, MSCs under the optimal preservation conditions described here harness diverse adaptations to maintain the functionality of these cells in response to the surrounding environment. Future studies are still required to explore the mechanism underlying our observations, especially with regard to metabolic alteration and paracrine secretion of these MSCs.

## CONFLICT OF INTEREST

The authors have no conflict of interest to declare.

## AUTHOR CONTRIBUTION

**Anh T.L. Ngo:** Data curation (equal); Formal analysis (equal); Methodology (equal); Resources (equal); Software (equal); Validation (equal); Visualization (equal); Writing‐original draft (equal); Writing‐review & editing (equal). **Hang M. Le:** Data curation (lead); Formal analysis (equal); Methodology (equal); Resources (equal); Software (equal); Validation (equal); Visualization (equal); Writing‐original draft (equal); Writing‐review & editing (equal). **Nhung T. H. Trinh:** Data curation (equal); Formal analysis (equal); Methodology (equal); Resources (supporting); Validation (supporting); Visualization (supporting); Writing‐original draft (supporting); Writing‐review & editing (supporting). **Adriel Peng Guo Jun:** Data curation (supporting); Formal analysis (supporting); Methodology (supporting); Resources (supporting); Validation (supporting); Visualization (supporting); Writing‐original draft (supporting); Writing‐review & editing (supporting). **Trung Q. Bach:** Data curation (supporting); Formal analysis (supporting); Methodology (supporting); Resources (supporting); Software (supporting); Validation (supporting); Writing‐original draft (supporting); Writing‐review & editing (supporting). **Hue T. H. Bui:** Formal analysis (supporting); Methodology (supporting); Validation (supporting). **Van T. Hoang:** Formal analysis (supporting); Methodology (supporting); Resources (supporting); Supervision (supporting); Validation (supporting); Writing‐original draft (supporting); Writing‐review & editing (supporting). **Anh Viet Bui:** Project administration (supporting); Resources (supporting); Supervision (supporting); Validation (supporting); Writing‐original draft (supporting); Writing‐review & editing (supporting). **Liem Thanh Nguyen:** Conceptualization (supporting); Funding acquisition (supporting); Project administration (supporting); Resources (supporting); Supervision (supporting); Validation (supporting); Writing‐original draft (supporting); Writing‐review & editing (supporting). **Duc M. Hoang:** Conceptualization (lead); Data curation (lead); Formal analysis (supporting); Funding acquisition (lead); Investigation (lead); Methodology (lead); Project administration (equal); Resources (lead); Software (equal); Supervision (lead); Validation (lead); Visualization (equal); Writing‐original draft (lead); Writing‐review & editing (lead).

## Supporting information

Fig S1

Fig S2

Fig S3

Table S1‐S3

## Data Availability

The data that support the findings of this study are available from the corresponding author upon reasonable request.
