# Supplementary figures and images for: Clinically relevant preservation conditions for mesenchymal stem/stromal cells derived from perinatal and adult tissue sources
Source: J Cell Mol Med. 2021 Oct 27;25(22):10747–60. doi: 10.1111/jcmm.17016 (PMC8581317; doi:10.1111/jcmm.17016)

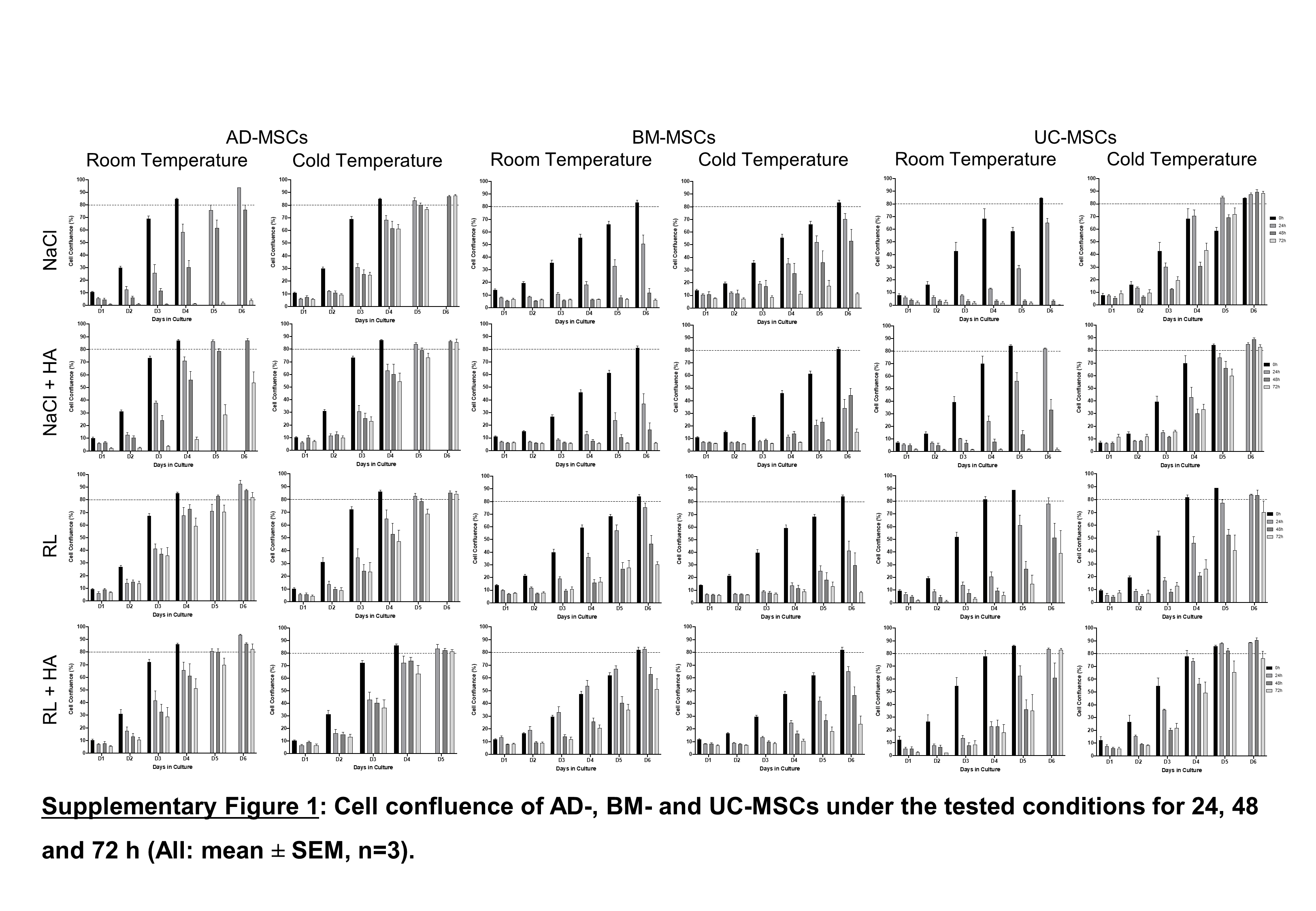

Supplement: Supplementary file 1 — Fig S1 [file JCMM-25-10747-s003.tif]

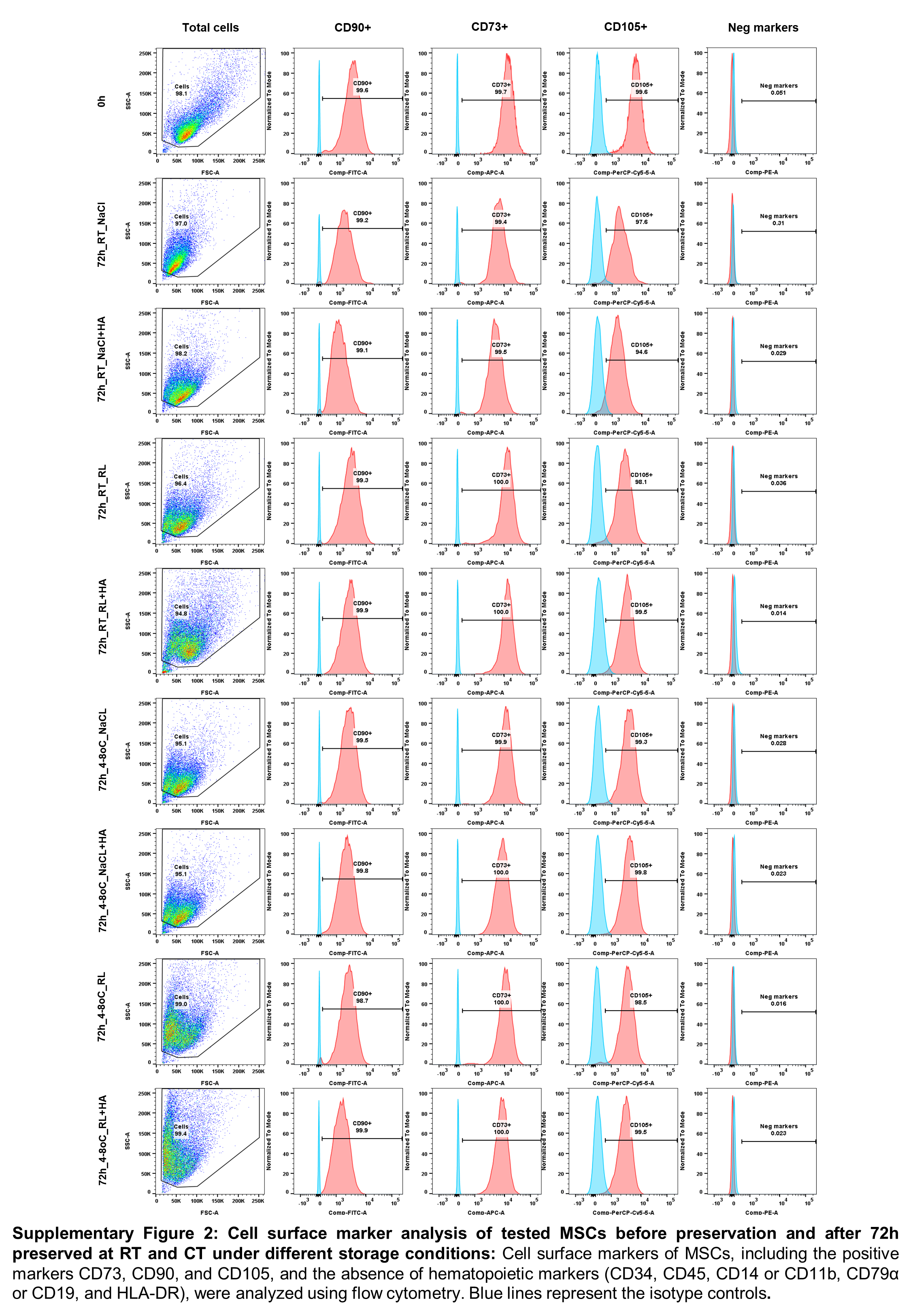

Supplement: Supplementary file 2 — Fig S2 [file JCMM-25-10747-s001.tif]

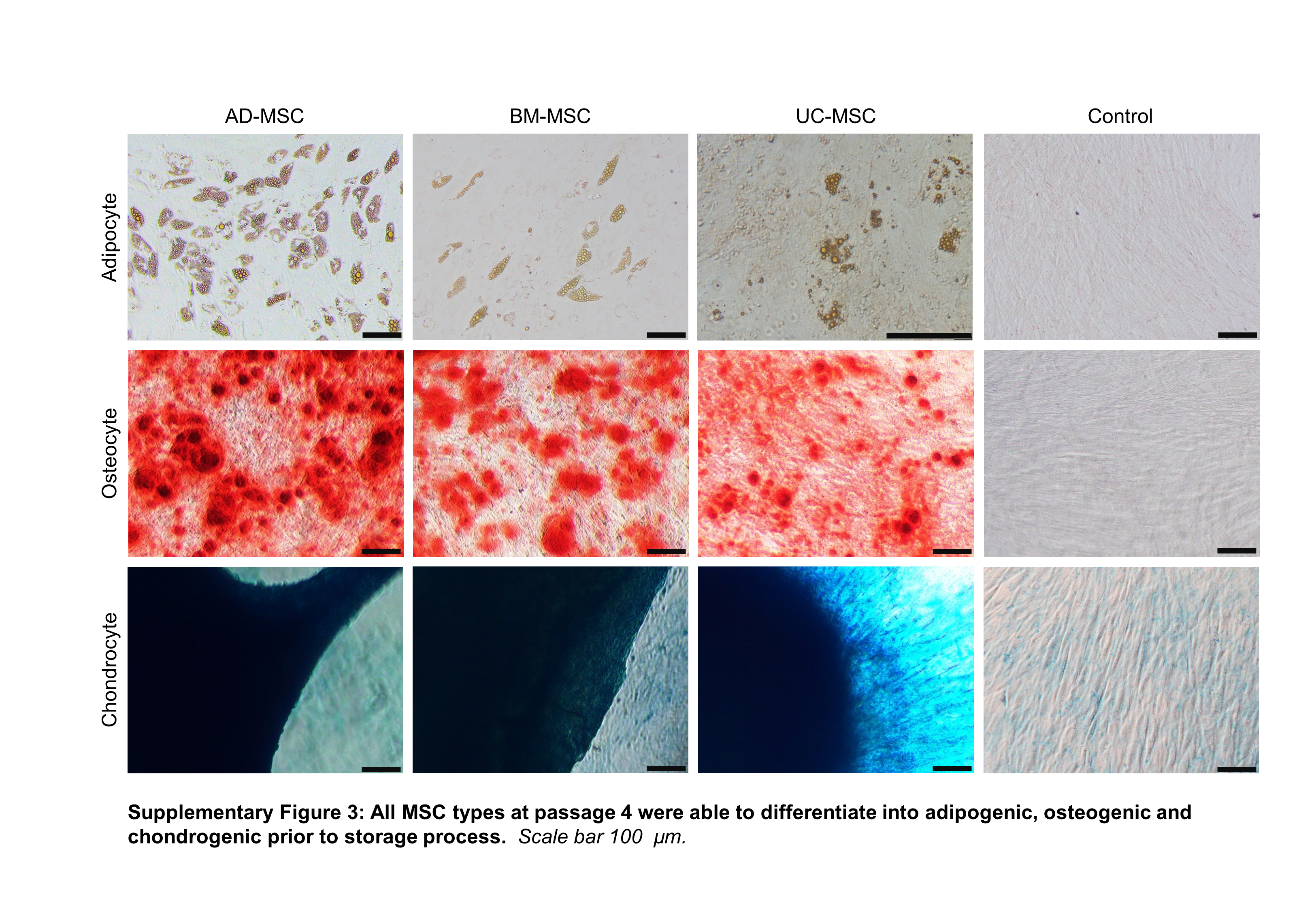

Supplement: Supplementary file 3 — Fig S3 [file JCMM-25-10747-s004.tif]
