# Supplementary material for: Clinically relevant preservation conditions for mesenchymal stem/stromal cells derived from perinatal and adult tissue sources
Source: J Cell Mol Med. 2021 Oct 27;25(22):10747–60. doi: 10.1111/jcmm.17016 (PMC8581317; doi:10.1111/jcmm.17016)
Supplement: Supplementary file 4 — Table S1‐S3 [file JCMM-25-10747-s002.docx]

**Supplementary Table 1: Viability of MSCs derived from AD, BM and UC under tested preservation conditions using Trypan Blue method. (All: mean ± SEM, n=3).**

| **Sample name** | **Preservation condition** | **Room temperature** | | | | **Cool temperature** | | | |
| --- | --- | --- | --- | --- | --- | --- | --- | --- | --- |
|  |  | **0h** | **24h** | **48h** | **72h** | **0h** | **24h** | **48h** | **72h** |
| **AD-MSCs** | **NaCl** | 89.78 ± 1.05 | 77.11 ± 2.46 | 70.33 ± 2.60 | 65.89 ± 3.05 | 89.78 ± 1.05 | 69.78 ± 5.49 | 62.44 ± 1.65 | 62.44 ± 3.28 |
|  | **NaCl+0.4%HA** | 93.89 ± 2.09 | 86.33 ± 1.39 | 72.44 ± 2.14 | 73.56 ± 4.74 | 93.89 ± 2.09 | 68.33 ± 4.00 | 40.00 ± 4.14 | 40.00 ± 1.92 |
|  | **RL** | 92.11 ± 1.21 | 88.22 ± 1.44 | 87.56 ± 1.71 | 84.22 ± 1.88 | 92.11 ± 1.21 | 85.56 ± 1.53 | 86.33 ± 1.96 | 86.33 ± 2.60 |
|  | **RL+0.4%HA** | 96.78 ± 1.13 | 94.00 ± 1.03 | 92.67 ± 0.83 | 86.33 ± 1.84 | 96.78 ± 1.21 | 93.56 ± 1.34 | 91.67 ± 1.33 | 91.67 ± 1.71 |
| **BM-MSCs** | **NaCl** | 75.11 ± 3.04 | 33.44 ± 5.07 | 26.33 ± 4.54 | 34.00 ± 5.31 | 75.11 ± 3.04 | 44.67 ± 3.82 | 13.00 ± 1.59 | 9.00 ± 2.27 |
|  | **NaCl+0.4%HA** | 88.89 ± 2.67 | 45.56 ± 6.01 | 18.67 ± 5.05 | 19.00 ± 2.08 | 88.89 ± 2.67 | 44.56 ± 1.86 | 8.67 ± 1.36 | 3.89 ± 1.01 |
|  | **RL** | 72.00 ± 3.83 | 67.44 ± 5.14 | 63.44 ± 3.86 | 59.78 ± 4.23 | 72.00 ± 3.83 | 73.78 ± 1.79 | 58.56 ± 1.29 | 58.56 ± 4.14 |
|  | **RL+0.4%HA** | 89.22 ± 1.91 | 83.33 ± 1.24 | 74.67 ± 1.04 | 69.89 ± 1.43 | 89.22 ± 1.91 | 82.33 ± 2.51 | 64.78 ± 1.60 | 59.78 ± 1.53 |
| **UC-MSCs** | **NaCl** | 82.00 ± 2.24 | 53.96 ± 4.73 | 36.83 ± 7.10 | 16.17 ± 2.89 | 82.00 ± 2.24 | 64.97 ± 0.70 | 56.00 ± 2.96 | 31.89 ± 2.65 |
|  | **NaCl+0.4%HA** | 91.38 ± 0.73 | 64.93 ± 3.68 | 54.20 ± 1.98 | 51.33 ± 1.54 | 91.38 ± 0.73 | 61.71 ± 5.48 | 34.67 ± 1.78 | 18.33 ± 1.60 |
|  | **RL** | 85.75 ± 2.50 | 61.98 ± 4.68 | 55.60 ± 4.37 | 52.50 ± 2.11 | 85.75 ± 2.50 | 78.80 ± 1.90 | 64.00 ± 1.47 | 50.89 ± 4.91 |
|  | **RL+0.4%HA** | 93.63 ± 0.63 | 73.20 ± 3.34 | 71.67 ± 3.15 | 67.00 ± 1.29 | 93.63 ± 0.63 | 87.44 ± 0.97 | 76.44 ± 1.86 | 67.22 ± 3.68 |

**Supplementary Table 2: Viability of MSCs derived from AD, BM and UC under the tested preservation conditions using flow cytometry analysis of 7-AAD staining. (All: mean ± SEM, n=3)**

| **Sample** | **Preservation condition** | **Room temperature (%)** | | | | **Cool temperature (%)** | | | |
| --- | --- | --- | --- | --- | --- | --- | --- | --- | --- |
|  |  | **0h** | **24h** | **48h** | **72h** | **0h** | **24h** | **48h** | **72h** |
| **AD-MSCs** | **NaCl** | 91.52 ± 1.04 | 84.92 ± 2.96 | 71.48 ± 4.44 | 73.72 ± 3.68 | 91.52 ± 1.04 | 83.59 ± 3.97 | 80.45 ± 3.50 | 71.18 ± 3.30 |
|  | **NaCl + HA** | 96.85 ± 0.67 | 87.87 ± 1.55 | 75.46 ± 2.26 | 66.49 ± 3.51 | 96.85 ± 0.67 | 76.28 ± 4.19 | 58.90 ± 1.93 | 48.49 ± 3.82 |
|  | **RL** | 88.31 ± 1.50 | 90.29 ± 1.94 | 87.96 ± 1.77 | 84.08 ± 2.67 | 88.31 ± 1.50 | 87.18 ± 2.15 | 89.19 ± 1.04 | 90.54 ± 1.39 |
|  | **RL + HA** | 97.58 ± 0.41 | 93.33 ± 0.72 | 89.34 ± 0.97 | 82.74 ± 2.09 | 97.58 ± 0.41 | 93.17 ± 0.88 | 90.84 ± 1.12 | 90.52 ± 1.39 |
| **BM-MSCs** | **NaCl** | 87.50 ± 2.69 | 57.47 ± 10.31 | 51.53 ± 9.62 | 45.87 ± 1.07 | 87.50 ± 2.69 | 71.50 ± 6.01 | 51.18 ± 3.20 | 41.22 ± 5.23 |
|  | **NaCl + HA** | 90.33 ± 1.86 | 63.27 ± 5.29 | 27.18 ± 6.09 | 35.45 ± 5.81 | 90.33 ± 1.86 | 57.63 ± 2.24 | 26.57 ± 1.87 | 20.73 ± 0.76 |
|  | **RL** | 90.98 ± 2.21 | 81.18 ± 2.69 | 68.28 ± 3.80 | 65.82 ± 1.03 | 90.98 ± 2.21 | 81.65 ± 2.29 | 75.25 ± 0.50 | 65.00 ± 1.98 |
|  | **RL + HA** | 89.40 ± 1.91 | 87.65 ± 0.34 | 69.28 ± 4.54 | 69.73 ± 1.20 | 89.40 ± 1.91 | 86.27 ± 1.16 | 73.75 ± 0.87 | 56.63 ± 3.90 |
| **UC-MSCs** | **NaCl** | 86.35 ± 1.37 | 64.06 ± 2.66 | 45.36 ± 7.25 | 29.96 ± 5.84 | 87.62 ± 0.71 | 77.78 ± 2.43 | 72.10 ± 2.42 | 65.58 ± 1.78 |
|  | **NaCl + HA** | 91.09 ± 1.47 | 61.26 ± 4.24 | 56.35 ± 8.31 | 44.00 ± 6.40 | 91.60 ± 1.35 | 71.80 ± 5.47 | 61.28 ± 2.70 | 52.11 ± 4.50 |
|  | **RL** | 87.98 ± 1.22 | 69.15 ± 3.74 | 58.79 ± 6.55 | 51.84 ± 7.48 | 89.09 ± 0.83 | 79.25 ± 2.77 | 80.09 ± 0.93 | 70.73 ± 1.94 |
|  | **RL + HA** | 91.50 ± 0.58 | 70.40 ± 3.07 | 62.66 ± 8.45 | 55.60 ± 7.87 | 92.26 ± 0.40 | 80.90 ± 2.38 | 82.66 ± 1.66 | 75.98 ± 0.48 |

**Supplementary Table 3: Pre- and post-preservation changes in growth factors and cytokines released from AD-, BM- and UC-MSCs (All: pg/ml**, **mean ± SEM, n=3)**

|  | **Sample name** | **AD-MSCs** | | | **BM-MSCs** | | | **UC-MSCs** | | |
| --- | --- | --- | --- | --- | --- | --- | --- | --- | --- | --- |
|  |  | **0h** | **7h Best** | **72h Worst** | **0h** | **7h Best** | **72h Worst** | **0h** | **7h Best** | **72h Worst** |
| **Growth factors** | **BDNF** | **-** | **-** | **-** | **-** | **-** | **-** | **-** | **-** | **-** |
|  | **LIF** | 9.06 ± 5.76 | **-** | **-** | 30.34 ± 7.79 | 46.19 ± 15.35 | 10.41 ± 10.41 | 121.42 ± 27.89 | **-** | **-** |
|  | **SCF** | **-** | **-** | **-** | **-** | **-** | **-** | **-** | **-** | **-** |
|  | **VEGF-D** | **-** | **-** | **-** | **-** | **-** | **-** | **-** | **-** | **-** |
|  | **bNGF** | **-** | **-** | **-** | **-** | **-** | **-** | **-** | **-** | **-** |
|  | **EGF** | **-** | **-** | **-** | **-** | **-** | **-** | **-** | **-** | **-** |
|  | **FGF-2** | **-** | **-** | **-** | **-** | **-** | **-** | **-** | **-** | **-** |
|  | **HGF** | 69.96 ± 29.60 | 103.18 ± 18.32 | 155.30 ± 9.23 | **-** | **-** | **-** | 29.83 ± 11.51 | **-** | **-** |
|  | **PDGF-BB** | **-** | **-** | **-** | **-** | **-** | **-** | **-** | **-** | **-** |
|  | **PIGF-1** | **-** | **-** | **-** | **-** | **-** | **-** | **-** | **-** | **-** |
|  | **VEGF-A** | 670.94 ± 210.54 | 586.73 ± 103.00 | 598.13 ± 100.61 | 904.04 ± 54.76 | 607.30 ± 31.27 | 721.14 ± 122.06 | **-** | **-** | **-** |
| **Cytokines** | **GM-CSF** | **-** | **-** | **-** | **-** | **-** | **-** | **-** | **-** | **-** |
|  | **IDO** | **-** | **-** | **-** | **-** | **-** | **-** | **-** | **-** | **-** |
|  | **IFN-gamma** | **-** | **-** | **-** | **-** | **-** | **-** | **-** | **-** | **-** |
|  | **IL-10** | **-** | **-** | **-** | **-** | **-** | **-** | **-** | **-** | **-** |
|  | **IL-1beta** | **-** | **-** | **-** | **-** | **-** | **-** | **-** | **-** | **-** |
|  | **IL-4** | 6.86 ± 4.35 | **-** | **-** | **-** | **-** | **-** | 19.89 ± 11.55 | **-** | **-** |
|  | **IL-6** | 294.24 ± 79.43 | 114.91 ± 57.53 | 73.33 ± 39.82 | 19.61 ± 12.41 | 38.80 ± 24.54 | 60.49 ± 38.31 | 191.11 ± 20.25 | 10.80 ± 6.46 | **-** |
|  | **IL-8** | 224.71 ± 103.81 | 226.76 ± 96.76 | 198.88 ± 62.19 | 83.70 ± 4.99 | 123.81 ± 14.97 | 174.92 ± 19.33 | 481.58 ± 221.97 | 88.19 ± 12.14 | **-** |
|  | **MCP-1** | 1097.64 ± 274.57 | 1303.91 ± 292.31 | 1267.49 ± 144.17 | 2103.26 ± 233.56 | 3201.89 ± 452.46 | 2467.69 ± 786.54 | 1407.70 ± 753.91 | 1033.07 ± 243.27 | **-** |
|  | **RANTES** | **-** | **-** | **-** | **-** | **-** | **-** | **-** | **-** | **-** |
|  | **TNF-alpha** | 8.85 ± 3.34 | **-** | **-** | **-** | **-** | **-** | **-** | **-** | **-** |
